# Thermal Degradation of Vegetable Oils

**DOI:** 10.3390/foods12091839

**Published:** 2023-04-28

**Authors:** Yi-Hsiou Tsai, Donyau Chiang, Yu-Ting Li, Tsong-Pyng Perng, Sanboh Lee

**Affiliations:** 1 Department of Materials Science and Engineering, National Tsing Hua University, Hsinchu 300, Taiwan; 2 National Applied Research Laboratories, Taiwan Instrument Research Institute, Hsinchu 300, Taiwan

**Keywords:** vegetable oil, optical absorbance, dynamic viscosity, electric impedance, acid value

## Abstract

Vegetable oils provide lipids and nutrition and provide foods with a desirable flavor, color, and crispy texture when used to prepare fried foods. However, the oil quality is degraded at elevated temperatures, and thus must be examined frequently because of the damage to human health. In this study, sunflower, soybean, olive, and canola oils were examined, and their properties were measured periodically at different elevated temperatures. The unsaturated triglyceride in oils reacted with the environmental oxygen or water vapor significantly changes in optical absorbance, viscosity, electrical impedance, and acid value. We used defect kinetics to analyze the evolution of these oil properties at elevated temperatures. The optical absorbance, viscosity, and electrical impedance follow the second-order, first-order, and zeroth-order kinetics, respectively. The rate constants of the above kinetics satisfy the Arrhenius equation. Olive oil has the lowest rate of color center and dynamic viscosity among the four oils, with the smallest pre-exponential factor and the largest activation energy, respectively. The rate constants of acid reaction also satisfy the Arrhenius equation. The activation energies of the polar compound and acid reaction are almost the same, respectively, implying that the rate constant is controlled by a pre-exponential factor if four oils are compared. Olive oil has the largest rate constant of acid reaction among the four oils, with the lowest pre-exponential factor.

## 1. Introduction

Frying is a food process in which the foods are immersed in hot oils from 150 °C to 200 °C to achieve the desired flavor, color, and crispy structure. Thus, oils play a significant role in frying, contributing to the nutrition, taste, smell, and texture of foods.

A major component in vegetable oils is triglycerides, which consist of three fatty acid chains connected to a glycerol backbone through the carboxyl group [1]. All the double bonds are positioned in the cis configuration in fresh vegetable oils. The reaction of oils during frying in air and water moisture is complicated. Chemical reactions, such as hydrolysis, oxidation, and polymerization [2], produce harmful compositions such as aldehyde, ketone, trans-fatty acid, and polycyclic aromatic hydrocarbons due to triglyceride oxidation during frying. In addition to causing foul odor and opaqueness, those harmful compounds have been reported to be relevant to eicosanoid metabolism, elevated oxidation stress [3], lipid and glucose metabolism, liver malfunction in rats [4], and potential for cancers in humans [5,6]. In addition to the chef’s experience in deciding whether the oils are discarded based on coloring, smoking, odd smells, or taste, we need instruments to determine the degradation of oils caused by heating.

Several instruments and methods are available to evaluate the quality of oil regarding its chemical compositions and physical properties. Fourier transform infrared spectroscopy (FTIR) with a database is a powerful instrument for identifying polar chemical compounds [7,8,9]. Chromatography with mass spectroscopy detects the quantity of total polar compounds of oils in vapor [10,11,12]. UV-vis spectrophotometry is another versatile instrument used to evaluate the compound change during frying via the color center [13,14,15]. As the thermal cycles of heating and cooling progress, the viscosity of oils increases due to the transformation of microstructure from unsaturated fat chains to long polymerization and blended chains. The viscosity meter effectively measures the change in the viscosity [16,17]. The titration determines the acid value of the fried oils, in which most governments enact standard values to discard deteriorated vegetable oils. In legislative regulation [13,14,15,16,17,18,19], the corrupted acid value ranges from 2 to 3 mg KOH/g, depending on the system of frying food and vegetable oil. However, the favored method reported in the literature [20,21,22] is an LCR meter with interdigital sensors of different geometries. The LCR measures electric impedance, capacitance, and dielectric constants, which are powerfully relevant to the degraded properties other than optical transmittance, viscosity, and acid value. A tested paper [19] and a hand-held device [23] are also commercially available to detect the acid value or total polar compounds in oils through color and digital monitoring, respectively. 

Using commercially available electronic components, Wang et al. made a cost-effective capacitance sensor [24]. The voltages obtained from their column chromatography measurements had a linear relationship with the total polar compounds of the tested edible oils. Yang et al. [25] summarized a table comparing the advantages and disadvantages of various methods used to characterize oil quality. However, no literature explained why the changes in optical transmittance, viscosity, and electric impedance of oils during heating, which prompted us to study the thermal degradation of vegetable oils. Four types of oil, including sunflower, soybean, olive, and canola, were analyzed. Based on the viewpoint of materials science, we explained the evolution of optical transmittance, viscosity, and electric impedance of vegetable oils using defect mechanism theory. The activation energies of the oils were obtained, and their acid values were evaluated.

## 2. Experimental Section 

Canola, sunflower, and olive oils were purchased from Taisun Enterprise Co. (Taipei City, Taiwan), and soybean oil from Taiwan Sugar Corp. (Hsinchu City, Taiwan). The oils were sealed and stored on a cool shelf to avoid exposure to the sun before the tests.

First, 125 mL of oil was poured into a 250 mL beaker in a silicon–oil bath to maintain a constant annealing temperature. The annealing temperatures were 140, 160, 180, and 200 °C, and the dwell time varied from 2 to 300 h, depending on the measurement method. The beaker was removed from the thermostatic bath and cooled to 25 °C for the test. When the test was not operated, the oil was stored in a refrigerator to slow the thermal degradation. 

The UV-visible transmittance of oils was measured on an ultraviolet-visible spectrophotometer (U-3900, Hitachi High-Technologies Co., Tokyo, Japan). Two cuvettes were used to correct the baseline. The baseline correction of wavelengths was scanned from 190 nm to 1300 nm with a scan rate of 300 nm/min. After finishing baseline correction, the oil-filled cuvette and empty cuvette are executed for the transmittance measurement. The scanning wavelength ranges from 300 nm to 800 nm, and the scanning speed is 300 nm/min. 

The dynamic viscosity of oils of 15 mL was determined at 25 °C on a 7990S Cannon-Ubbelohde glass viscometer (Ramin Corp., Magnolia, TX, USA). The reported viscosities are average values of five measurements. The measurement of the electrical impedance of heated oils was performed on an 8110G LCR meter (Good Well Electrics Industrial Co., New Taipei City, Taiwan) with a homemade interdigital sensor (see Reference [26] for the construction of the interdigital sensor). 

A cosolvent of 25.0 mL ethanol (Taiwan Sugar Corp., Tainan City, Taiwan) and 25.0 mL diethyl ether (Echo Chemical Co., Miaoli, Taiwan) was prepared as a blank titration solvent to dissolve the oil. The oil of 10.0 g was mixed with 50.0 mL blank solvent. A few drops of phenolphthalein (Echo Chemical Co., Miaoli, Taiwan) were added to the mixed solution as an indicator. The transparent base titrant was 0.010 M KOH. The titration was ended after the color of the solution changed to pink for more than 30 s. The acid value was calculated using Equation (1) below.

(1) $$\mathrm{Acid value}=\frac{(\Omega -\Omega_{0})\times C\times M}{W}$$

where Ω is the volume of the consumed titrant KOH after the titration, Ω_0_ is the volume of titrant KOH for a blank titration solvent, *C* is the concentration of the base titrant (0.010 mol/L), *M* is the molecular weight of titrant (56.1 g/mol), and W is the oil weight (10.0 g).

The chemical bonds of heated oils were analyzed on a Perkin Elmer Spectrum 100 FTIR (Perkin Elmer Co., Shelton, CT, USA) with an attenuated total reflection mode. The scanning wavenumber range was from 450 to 4000 cm^−1^.

The experimental data of ultraviolet-visible spectroscopy, dynamic viscosity, electrical impedance, and acid values are analyzed using the least square method. The least square method with confidence interval R^2^ was provided by the graphical software Origin (Electrical Art Inc., Redwood City, CA, USA).

## 3. Results and Discussion

### 3.1. Ultraviolet-Visible Spectroscopy

**Figure 1:**
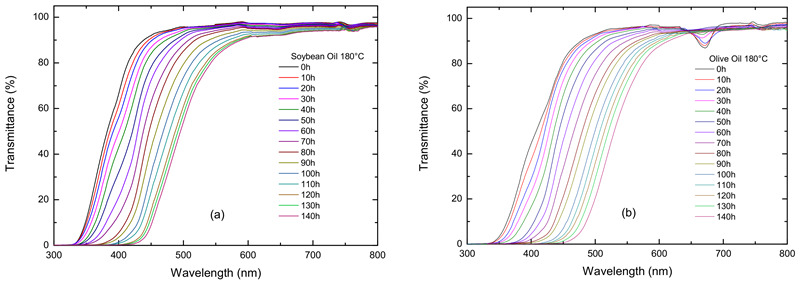
The variation of transmittance with the wavelength at 180 °C for different times: (**a**) soybean oil and (**b**) olive oil.

Figure 1a shows the transmittance spectra for soybean oil maintained at 180 °C at different times. The transmittance spectra exhibit S-like behavior with slow increase initially, rapid increase intermediately, and slow increase with increasing wavelength to reach a plateau. Increasing the heating time causes the right shift of the S-like spectrum. The transmittance spectra of the soybean oils heated at 140, 160, and 200 °C are presented in Figure S1a–c in Supplementary Information, respectively. According to Figure 1a and Figure S1a–c, the time to reach the steady-state transmittance decreases with increasing the heating time, implying that the evolution of transmittance is associated with a thermally activated process. A similar trend for the transmittance spectra is observed for canola, sunflower, and olive oils, as shown in Figures S2a–d, S3a–d, and S4a–c in Supplementary Information, respectively. The small peak for the wavelength at 670 nm observed in the transmittance spectra of the olive oil, as shown in Figure 1b and Figure S4a–c, is attributed to chlorophyll [14,27], whose peak increases with increasing time and temperature because chlorophyll is thermally unstable.

**Figure 2:**
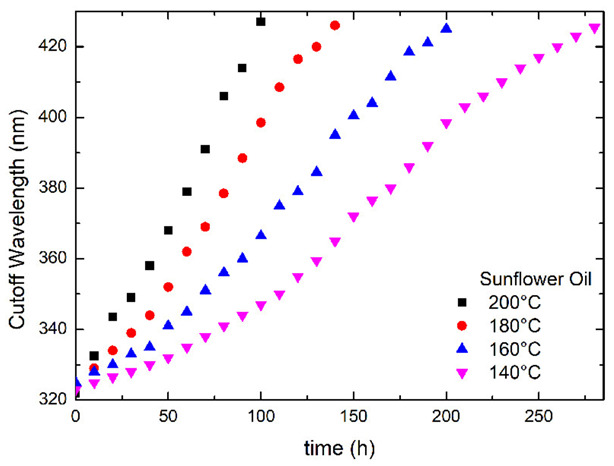
The relationship between the cutoff wavelength and times for the sunflower oil at different temperatures.

Define the cutoff wavelength as the maximum wavelength without transmittance. Figure 2 shows the temporal evolution of the cutoff wavelength for the sunflower oil at different temperatures, obtained directly from Figure 1a and Figure S3 in the Supplementary Material. The cutoff wavelength exhibits a redshift with increasing time and temperature. Similar behavior is observed for the cutoff wavelengths of the soybean, canola, and olive oil, as shown in Figure S5 in Supplementary Material, and are 320 nm to 440 nm. Canola oil has the largest cutoff wavelength.

**Figure 3:**
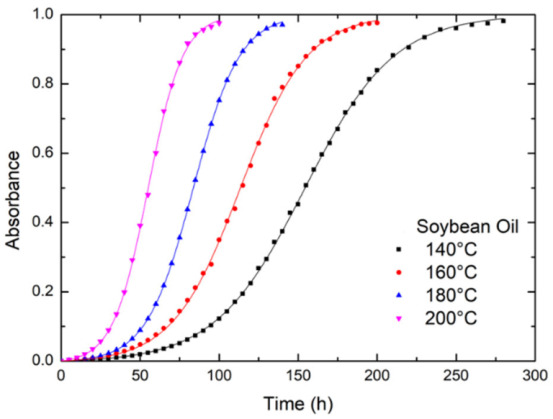
The variation of absorbance (A − A_0_) of soybean oil at 445 nm with annealing time at different temperatures. The solid lines are obtained using Equation (4).

From the viewpoint of energy conservation, the summation of reflectance (I_R_), absorbance (A), and transmittance (I_T_) is equal to unity. [28] Since the incident UV-visible light is normal on the specimen surface, the reflectance is negligible. Additionally, we are interested in the difference between absorbances at the initial state and the state after the heating for the dwell of t, which is equal to the difference between the corresponding two transmittances if other conditions are maintained at the same level. That is, the non-zero reflectance has a negligible effect on the absorbance difference. Figure 3 shows the variation in the absorbance difference (A − A_0_) at the wavelength of 445 nm with the heating time for the soybean oil heat-treated at 140, 1620,180, and 200 °C. Here, A_0_ and A are the absorbances at the initial state and the heating time t. Note that the absorbance A is equal to (1−I_T_), which is different from that obtained using the Lambert–Beer law (i.e., A= −Log I_T_). For each curve, the slope increases slowly initially, reaches constant, and then decreases to a plateau with the increasing heating time. The higher the temperature, the shorter the time to the plateau. Similar behavior is observed for other oils, as shown in Figures S6a–c in the Supplementary Information. At the same time, the absorbance increases with increasing temperature for all the oils.

Assume that the evolution of absorbance is controlled by the generation of color centers, which second-order kinetics can describe. The differential equation for second-order kinetics is expressed as

(2) $$\frac{dn}{dt}=\alpha_{A}\left(n_{\infty }-n\right)\left(1-\beta \left(n_{\infty }-n\right)\right)$$

where *n* is the concentration of color centers, *n*_∞_ is the concentration at infinite time, *α_A_* is the rate constant, *β* is the proportional constant related to absorbance, and t is the elapsed time. Note that the maximum absorbance is unity. The solution of Equation (2) with the initial condition, *n* = *n*_0_ at *t* = *t*_0_, is given as

(3) $$\frac{n_{\infty }-n}{n_{\infty }-n_{0}}=\frac{e^{-\alpha_{A}t}}{1+\beta (n_{\infty }-n_{0})\left(e^{-\alpha_{A}t}-1\right)}$$

Since *A* = *βn* and the initial condition *A* = *A*_0_ at *t* = 0, Equation (3) is rearranged as

(4) $$\frac{A-A_{0}}{A_{\infty }-A_{0}}=1-\frac{e^{-\alpha_{A}t}}{1+{(A}_{\infty }-A_{0})\left(e^{-\alpha_{A}t}-1\right)}$$

For the soybean oils, the solid lines in Figure 3 are obtained using Equation (4) with parameters listed in Table 1. Similarly, the parameters for the other oils used in Equation (4) fit the absorbance data in Figure S6a–c are shown in Tables S1–S3 in Supplementary Material, respectively. The rate constant of every vegetable oil increases with the annealing temperature.

**Figure 4:**
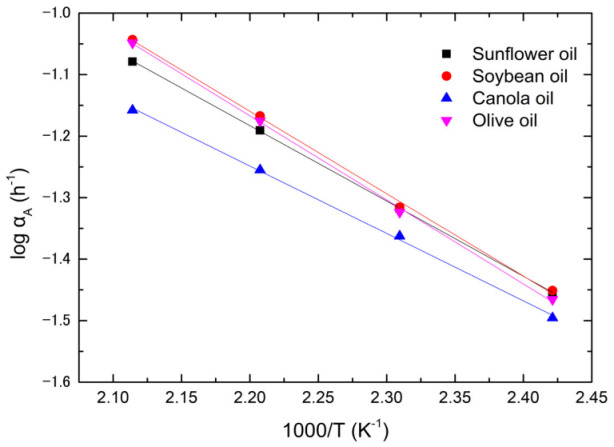
The log (α_A_) plots versus 1000/T for sunflower, canola, soybean, and olive oils.

**Figure 5:**
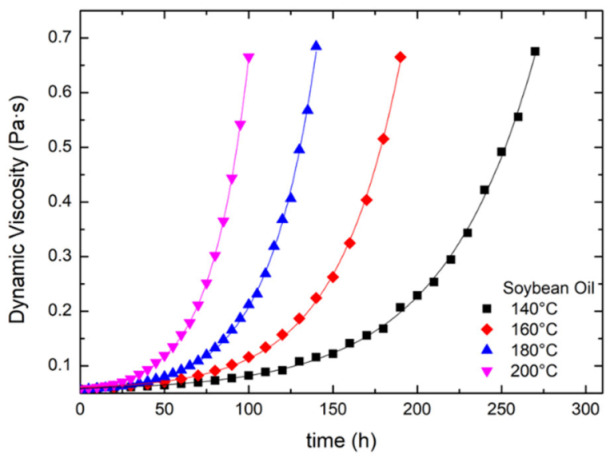
The variations of dynamic viscosity with the annealing time for soybean oil at different temperatures.

The logarithm plot of the rate constant with the reciprocal absolute temperature is shown in Figure 4. According to Figure 4, the rate constant satisfies the Arrhenius equation,

(5) $$\alpha_{A}=\alpha_{A0}\exp\left(\frac{-Q_{A}}{RT}\right)$$

where *α_A_*_0_ is the pre-exponential factor, *Q_A_* is the activation energy, *R* is the gas constant, and *T* is the Kelvin temperature. The activation energy and pre-exponential factor of absorbance for all oils are tabulated in Table 2. Canola oil has the largest pre-exponential factor and activation energy among the four types of oil. The descending activation energy of four types of vegetable oils is sunflower oil, canola oil, soybean oil, and olive oil, respectively. The higher the activation energy, the more resistance the chemical reaction induces to the color center and optical absorbance. However, the pre-exponential factor also plays a vital role in color center kinetics, which is a trade-off for the activation energy. Comparing Table 2 and Figure 5, canola oil has the lowest rate constant and largest pre-exponential factor and activation energy among the four oils, implying that the rate constant is dominated by activation energy. Olive oil has the greatest rate constant and smallest pre-exponential factor and activation energy of optical absorbance, suggesting that activation energy controls the rate constant.

### 3.2. Dynamic Viscosity

The variations of dynamic viscosity with the annealing time for soybean oils at different temperatures are exhibited in Figure 5. A similar trend is found for the other three types of vegetable oils, as shown in Figure S7a–c in the Supplementary Material. The dynamic viscosity increases exponentially with the annealing time at a given temperature, implying the defects hindering the oil flow. The higher the temperature, the shorter time needed for the oil to arrive at a given viscosity, e.g., 0.5 Pa·s.

The defect hindering the molecular motion is assumed to contribute to the dynamic viscosity. The defect concentration, *n_v_*, follows the first-order generation kinetics as shown below

(6) $$\frac{dn_{v}}{dt}=\alpha_{v}n_{v}$$

where *α_v_* is the rate constant of dynamic viscosity and *t* is the annealing time. The solution of Equation (6) with the initial concentration, *n_v_*_0_, at *t* = 0 is obtained as

(7) $$n_{v}=n_{v0}\exp\left(\alpha_{v}t\right),$$

Further, we assumed that the total dynamic viscosity *φ* consists of a thermally independent component, *φ_R_*, and the thermal component, *φ* − *φ_R_*. The thermal component of dynamic viscosity is linearly proportional to the defect concentration as *φ* − *φ_R_* = *βn_v_,* where β is a constant. Using this relation, Equation (7) is rearranged as

(8) $$\varphi =\varphi_{R}+\left(\varphi_{0}-\varphi_{R}\right)e^{\alpha_{v}t}$$

The solid lines in Figure 5 for the soybean oil are obtained using Equation (8) with the parameters *φ*_0_, *φ_R_*, and *α_v_* listed in Table 3. The corresponding parameters used for the other three vegetable oils are in Tables S4–S6 in the Supplementary Material, respectively. According to Table 3 and Tables S4−S6 in the Supplementary Material, olive and canola oils are the first two largest *φ*_0_ and *φ_R_* among the four oils, likely attributed to chlorophyll and carotene [14,27].

**Figure 6:**
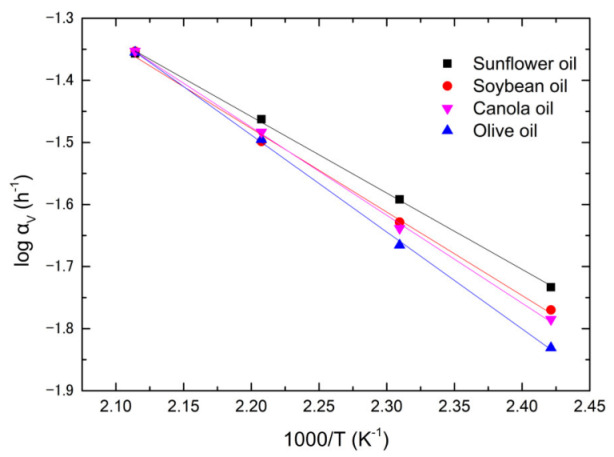
The Arrhenius plots of dynamic viscosity for four vegetable oils.

Using Table 3 and Tables S4–S6 in Supplementary Material, the plots of Log *α_v_* versus 1/*T* for the soybean, canola, olive, and sunflower oils are shown in Figure 6. The rate constants, *α_v_*, are fitted by the following Arrhenius equation,

(9) $$\alpha_{v}=\alpha_{v0}\exp\left(\frac{-Q_{v}}{RT}\right)$$

where *α_v_*_0_ and *Q_v_* are the pre-exponential factor and activation energy of dynamic viscosity, respectively. *R* and *T* follow the conventional definition. The experimental data are in good agreement with the theoretical prediction. The corresponding activation energies and pre-exponential factors for the four types of vegetable oils are tabulated in Table 4. The oil with a higher pre-exponential factor possesses a greater amount of activation energy. According to Equation (9), the rate constant increases with the increasing pre-exponential factor and decreasing activation energy, implying that the pre-exponential factor is a trade-off for the activation energy. Comparing Figure 6 and Table 4, the lower the rate constant, the greater the activation energy and pre-exponential factor. Olive oil has the lowest rate constant and largest activation energy and pre-exponential factor of dynamic viscosity, implying that the evolution of dynamic viscosity is controlled by the activation energy. Similarly, sunflower oil has the greatest rate constant and smallest pre-exponential factor and activation energy among the four oils, implying that the evolution of dynamic viscosity is controlled by thermally activated defect kinetics.

### 3.3. Electrical Impedance

**Figure 7:**
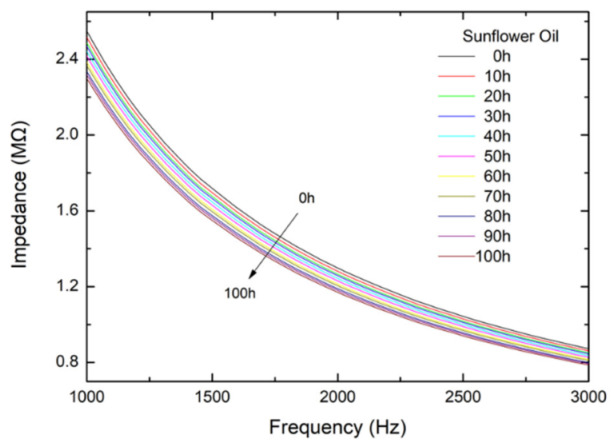
The impedance variations with frequency for sunflower oil annealed at 200 °C at different times.

The vegetable oils are dielectric materials, and their electrical impedances are measured by an LCR meter with an interdigital sensor of 100 µm. The frequency range is selected from 1000 Hz to 3000 Hz. The relations between electrical impedance and frequency for sunflower oil under the applied voltage of 1 V at 200 °C are exhibited in Figure 7. The variations in impedance with frequency for the other three oils annealed at 200 °C and 160 °C for the different times are displayed in Figures S8 and S9 in the Supplementary Material. The impedance decreases with the increase in the annealing time and frequency for every oil. The impedance changing from *t* = 0 to 100 h at the given frequency is the largest for sunflower oil, followed by soybean oil, olive oil, and canola oil.

**Figure 8:**
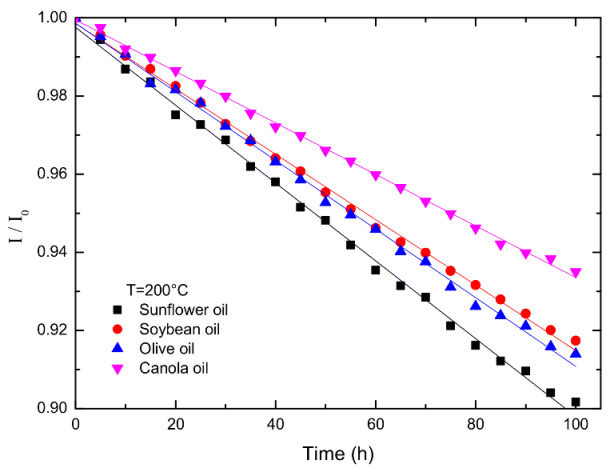
The variations in impedance versus annealing time for the four types of oil annealed at 200 °C.

From Figure 7 and Figure S8 in Supplementary Material, we plotted the impedance versus annealing time for the four oils measured at 1000 Hz and annealed at 200 °C in Figure 8, where *I*_0_ is the initial impedance. Note that the initial impedances are listed in Table S7 in Supplementary Material, which was extrapolated from the impedances of the short times at 1000 Hz. The initial impedances are almost the same for all types of oil and the annealing temperatures. Figure S10a–c show the variations in impedance with annealing time for oils measured at 1000 Hz and annealed at (a) 180 °C, (b) 160 °C, and 140 °C, respectively. The impedance decreases linearly with the annealing time. Sunflower oil has the steepest slope, and canola oil has the smallest one.

Assume that the positive charges (or negative charges) of electric dipole in the oil consist of two components: one arises from thermal annealing, *P_A_*, and the other is generated from sources other than thermal annealing, *P*_0_. Then, the positive charges *P* are the sum of *P_A_* and *P*_0_. Further, assume that *P_A_* is linearly proportional to the concentration of defects carrying charges, i.e., *P_A_* = *βn_E_*, in which *β* and *n_E_* are the proportional constant and defect concentration, respectively. The defect concentration contributing to the induced charges follows the zeroth-order kinetics, $dn_{E}/dt=\alpha_{1}.$ The solution of this equation with the initial free concentration is *n_E_* = *α*_1_*t*. The positive charges can be written as *P* = *P*_0_ + *βα*_1_*t*= *P*_0_ + *β_E_t* where *β_E_* is the charge rate constant. The electrical impedance is expressed as $I=\sqrt{r^{2}+1/\omega^{2}C^{2}}$, where *r*, *ω*, and *C* are resistance, angular frequency, and capacitance, respectively. Substituting $C=P/V={(P}_{0}+\beta_{E}t)/V$ into the above equation, we have $I=\sqrt{r^{2}+V^{2}/\omega^{2}(P_{0}+\beta_{E}t{)}^{2}}$. Note that V is the applied voltage. Using Taylor’s series expansion to the first-order approximation, the impedance is as follows,

(10) $$\frac{I}{I_{0}}=\frac{\sqrt{r^{2}+\frac{V^{2}}{\omega^{2}P^{2}}}}{\sqrt{r^{2}+\frac{V^{2}}{\omega^{2}P_{0}^{2}}}}\sim 1-\frac{\beta_{E}t}{{(1+r}^{2}\omega^{2}P_{0}^{2}/V^{2})P_{0}}=1-\alpha_{E}t$$

Since *r*, *ω*, *V*, and *P*_0_ are temperature-independent parameters, $\alpha_{E}$ is a rate constant and satisfies the Arrhenius equation,

(11) $$\alpha_{E}=\alpha_{E0}\exp\left(-Q_{E}/RT\right)$$

where *Q_E_* and $\alpha_{E0}$ are the activation energy and pre-exponential factor of the impedance of the degraded oils, and R and T have their ordinary meanings.

**Figure 9:**
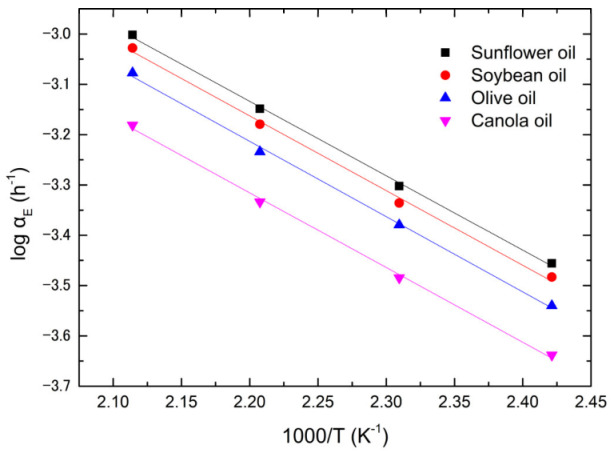
The Arrhenius plots of the impedance for four types of vegetable oils.

The plots of the logarithm of the rate constant versus the reciprocal absolute temperature for different oils are shown in Figure 9. Using Equation (11) to fit the data in Figure 9, one obtains the pre-exponential factors and activation energies and lists them in Table 5. The activation energies of all oils are the same within the error bar, implying that the energy barriers for the polar compound reaction are identical for all oils. Comparing Figure 9 and Table 5, the rate constant of impedance increases with the increase in the pre-exponential factor. The oils with decreasing pre-exponential factors of the polar compound are sunflower, soybean, olive, and canola oils. Since the pre-exponential factor is time independent, sunflower oil always has the largest rate constant of impedance, and canola oil has the smallest.

### 3.4. Acid Value

The vegetable oils exposed to the harsh environment of high temperature for a long duration can generate free fatty acid and glycerin. The triglyceride in oils absorbs and reacts with the water vapor in the air, and its hydrolysis reaction is described as

C_3_H_5_(COOR)_3_ + 3H_2_O → C_3_H_5_(OH)_3_ + 3RCOOH

**Figure 10:**
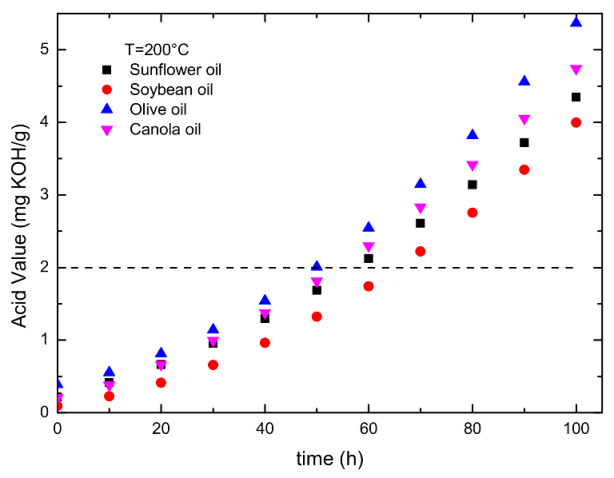
The variations of the acid value with annealing time for the oils annealed at 200 °C.

The higher the amount of free fatty acid in the oil, the worse the degraded oil, and the more consumable the titrant solution to neutralize the free acid. The variations in the acid value versus the time for the oils annealed at 200 °C are illustrated in Figure 10. The plots of acid value versus annealing time for all oils annealed at 180 °C, 160 °C, and 140 °C are shown in Figure S11a–c of the Supplementary Material, respectively. The acid value increases slowly in the beginning, then accelerates with the increase in the annealing time for all oils. Olive oil produces the easiest acid value, and soybean oil is the most difficult among the four types of oil.

**Figure 11:**
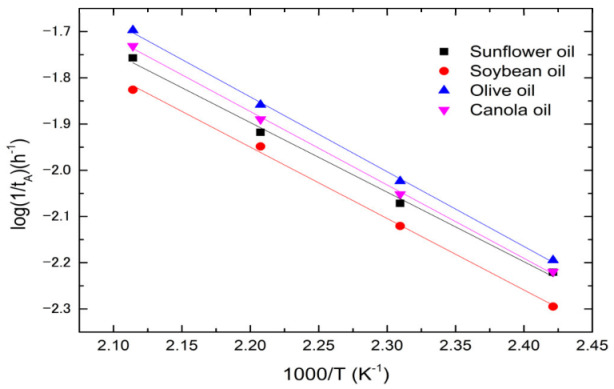
The log (1/*t_A_*) variations with (1/*T*) for all oils.

According to the oil rancidification generation and legislation regulation [18,19], when the acid value of vegetable oil exceeds 2.0 mg KOH/g, the oil is discarded. Let *t_A_* be the time when the oil reaches the acid value of 2.0 mg KOH/g. One obtains t_A_ from Figure 10 and Figure S11 in Supplementary Material and illustrates them in Figure 11. The rate constant of acid reaction equals 1/*t_A_*. Figure 11 shows the log (1/*t_A_*) plots versus (1/*T*) for all oils. It can be seen from Figure 11 that 1/*t_A_* satisfies the Arrhenius equation, i.e., $\frac{1}{t_{A}}=\left(\frac{1}{t_{A0}}\right)\exp\left(-\frac{Q_{AC}}{RT}\right),$ where *t_A_*_0_ and *Q_AC_* are the pre-exponential factor and activation energy for the acid reaction, and *R* and *T* are the gas constant and absolute temperature, respectively. Note that the definition of pre-exponential factor for the acid reaction is different from those for color center, dynamic viscosity, and polar compound. According to the above equation, the rate constant of an acid reaction is inversely proportional to the pre-exponential factor *t_A_*_0_ and activation energy. Table 6 lists the *t_A_*_0_ and activation energies of acid reaction for all oils obtained from Figure 11. The activation energies for olive, canola, soybean, and sunflower oil are almost equal within ±1.0 kJ/mol. Comparing Table 6 and Figure 11, the pre-exponential factor independent of temperature controls the acid reaction. The oils with increasing pre-exponential factor t_A0_ of acid reaction are olive, canola, sunflower, and soybean. Olive oil has the largest rate constant because its *t_A_*_0_ is the smallest one.

### 3.5. Fourier Transform Infrared Spectroscopy (FTIR)

**Figure 12:**
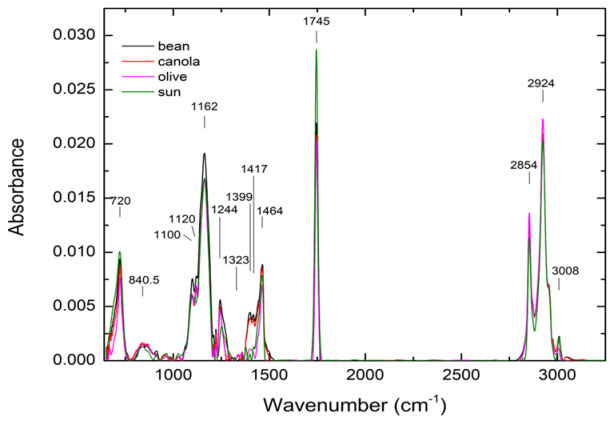
The FTIR spectra of all oils without heat treatment.

The FTIR spectra of all oils without heat treatment are shown in Figure 12, with their chemical bonds listed in Table 7. Because triglyceride is a significant component in all vegetable oils, the spectral features of the FTIR spectroscopy look very similar, with slight minor differences in peak height and width to reflect the unique character of each oil type. The major peaks at wavenumbers 720, 1110, 1120, 1162, 1244, 1399, 1464, 1745, 2854, 2924, and 3008 cm^−1^ coincided for all oils, implying that the oils exhibit the same molecular structure reported in references [9,28].

## 4. Conclusions

Edible oil quality is essential to human health. Soybean, canola, olive, and sunflower oils are studied. The UV-vis transmittance, chemical acid value (titration), dynamic viscosity, and electrical impedance measurements are used to evaluate the oil degradation at elevated temperatures. The cutoff wavelength is redshift, and its shifting rate increases with the increase in annealing time. The curves of the optical absorbance at a given wavelength versus annealing time grow slowly at short times, rapidly at intermediate times, and slowly again until they approach a plateau at long times. The dynamic viscosity of the vegetable oils increases exponentially with the annealing time at a given temperature. The electric impedance decreases with the frequency and annealing time. The acid value increases exponentially in the early stage and then keeps a roughly constant rate. We propose second-order, first-order, and zeroth-order kinetics to describe the evolutions of optical absorbance, dynamic viscosity, and electrical impedance. The rate constants satisfy the Arrhenius equation. Olive oil has the lowest rates of color centers and dynamic viscosity among the four oils because it has the smallest pre-exponential factor for the former and the largest activation energy for the latter, respectively. The reciprocal of time to reach acid value also satisfies the Arrhenius equation. The activation energies of the polar compound and acid reaction, respectively, are identical for all oils to reflect the same evolution path. Olive oil has the largest rate constant among the four oils with the smallest pre-exponential factor, *t_A_*_0_.

## Figures and Tables

**Table 1:** Parameters *α_A_*, *A*_0_, and *A*_∞_ used in Equation (4) to fit the absorbances of soybean oil at four annealing temperatures where *R*^2^ is the confidence interval.

Temperature (°C)	*α_A_* (h^−1^)	*A*_0_	*A*_∞_	*R* ^2^
200	0.091	0.0362	0.9632	0.998
180	0.068	0.0852	0.9951	0.997
160	0.047	0.0867	0.9774	0.996
140	0.035	0.0321	0.9675	0.997

**Table 2:** The activation energies and pre-exponential factors of optical absorbance for four types of oils.

Oil Type	*α_A_*_0_ (h^−1^)	*Q_A_* (kJ/mol)	*R* ^2^
Soybean	58.34	25.44	0.995
Canola	69.18	26.17	0.994
Olive	14.58	20.99	0.998
Sunflower	31.98	23.40	0.997

**Table 3:** The parameters *φ*_0_, *φ_R_*, and *α_v_* yield the solid lines in Figure 6 for the soybean oil.

Annealing Time (°C)	*φ*_0_ (10^−2^ Pa·s)	*φ_R_* (10^−2^ Pa·s)	*φ*_0-_*φ_R_*(10^−2^ Pa·s)	*α_v_* (10^−2^ h^−1^)	*R* ^2^
200	5.743	5.015	0.728	4.434	0.993
180	5.208	4.455	0.753	3.476	0.992
160	5.377	4.582	0.795	2.625	0.991
140	5.648	5.011	0.637	1.699	0.998

**Table 4:** The activation energies and pre-exponential factors of viscosity for four types of oils.

Oil Type	*α_v_*_0_ (h^−1^)	*Q_v_* (kJ/mol)	*R* ^2^
Olive oil	88.7	29.90	0.996
Canola oil	43.8	27.14	0.997
Soybean oil	30.4	25.77	0.996
Sunflower oil	17.8	23.58	0.998

**Table 5:** The pre-exponential factor and activation energy of electrical impedance for four types of oils.

Oil Type	$\alpha_{E0}$	*Q_E_* (kJ/mol)	*R* ^2^
Soybean oil	1.27	28.44 (±0.53)	0.997
Canola oil	0.90	28.44 (±0.49)	0.998
Olive oil	1.20	28.66 (±0.58)	0.995
Sunflower oil	1.32	28.32 (±0.45)	0.997

**Table 6:** The activation energies and t_A0_ of the acid reaction for four oils.

Oil Type	*t_A_*_0_ (h)	*Q_AC_* (kJ/mol)	*R* ^2^
Olive	0.0192	30.9	0.997
Canola	0.0239	30.4	0.996
Sunflower	0.0369	29.0	0.995
Soybean	0.0392	29.1	0.996

**Table 7:** The peak positions with their chemical bonds.

Wavenumber (cm^−1^)	Description
3008 cm^−1^	C-H stretching vibration of the *cis*-double bond (=CH)
2924 cm^−1^, 2854 cm^−1^	Symmetric and asymmetric stretching vibration of the aliphatic CH_2_ group
1745 cm^−1^	Ester carbonyl functional group of the Triglycerides
1464 cm^−1^	Bending vibrations of the CH_2_ and CH_3_ aliphatic groups
1417 cm^−1^	Rocking vibrations of CH bonds of *cis*-disubstituted olefins
1399 cm^−1^	Bending in-ddplane vibrations of CH *cis*-olefinic groups
1323 cm^−1^	The extra peak appears in mixed oil.
1244 cm^−1^, 1162 cm^−1^, 1100 cm^−1^	Stretching vibration of the C-O ester Groups
1120 cm^−1^	Aliphatic ethers
840.5 cm^−1^	trisubstituted alkenes
720 cm^−1^	Overlapping of the CH_2_ rocking vibration and the out-of-plane vibration of *cis*-disubstituted olefins

## Data Availability

Not applicable.

## Supplementary Materials

The following supporting information can be downloaded at: https://www.mdpi.com/article/10.3390/foods12091839/s1, Figures S1–S4: UV-visible transmittance vs. wavelength for soybean, canola, sunflower, and olive oils, respectively. Figure S5: cutoff wavelengths, Figure S6: UV-visible absorbance, Figure S7: dynamic viscosity, Figures S8 and S9: electrical impedances vs. frequency at 200 and 160 °C, respectively. Figure S10: impedance vs. annealing time at frequency 1000 Hz. Figure S11: acid value vs. time. Tables S1–S3: optical absorbance parameters *α*, *A*_0_, and *A*_∞_ for canola, olive, and sunflower, respectively, Tables S4–S6: viscosity parameters *φ*_0_, *φ_R_*, and *α_v_* for canola, olive, and sunflower oils, respectively, Tables S7: oil initial impedances at frequency 1000 Hz.
