# Supplementary material for: Thermal Degradation of Vegetable Oils
Source: Foods. 2023 Apr 28;12(9):1839. doi: 10.3390/foods12091839 (PMC10178358; doi:10.3390/foods12091839)
Supplement: Supplementary file 1 [file foods-12-01839-s001.zip › foods-2319480-supplementary.pdf]

Supplementary Material

Thermal Degradation of Vegetable Oils

Yi-Hsiou Tsai[1], Donyau Chiang[2], Yu-Ting Li[2], Tsong-Pyng Perng[1], Sanboh Lee[1*]

[1] Department of Materials Science and Engineering, National Tsing Hua University, Hsinchu 300, Taiwan

[2] Taiwan Instrument Research Institute, National Applied Research Laboratories, Hsinchu 300, Taiwan

Figure Captions

Figure S1. The variations of UV-Vis transmittance versus wavelength for oils annealed for different times at temperatures: (a) 200°C, (b) 160°C, and (c) 140°C

Figure S2. The variations of UV-Vis transmittance versus wavelength for canola oils annealed for different times at temperatures: (a) 200°C, (b) 180°C, (c) 160°C, and (d) 140°C

Figure S3. The variations of UV-Vis transmittance versus wavelength for sunflower oils annealed for different times at temperatures: (a) 200°C, (b) 180°C, (c) 160°C, and (d) 140°C.

Figure S4. The variations of UV-Vis transmittance versus wavelength for mixed oils annealed for different times at temperatures: (a) 200°C, (b) 160°C, and (c) 140°C

Figure S5. The variations of cutoff wavelength with annealing time at different annealing temperatures for (a) soybean oil, (b) canola oil, and (c) olive oil.

Figure S6. The variations of absorbance with time at different annealing temperatures: (a) soybean oil, (b) olive oil, and (c) sunflower oil.

Figure S7. The variations of dynamic viscosity with annealing time at different annealing temperatures: (a) canola oil, (b) olive oil, and (c) sunflower oil.

Figure S8. The variations of electrical impedance with the frequency for (a) soybean oil, (b) canola oil, and (c) olive oil annealed at 200°C for different annealing times.

Figure S9. The variations of electrical impedance with the frequency for (a) soybean oil, (b) canola oil, (c) olive oil, and (d) sunflower oil annealed at 160°C for different annealing times.

Figure S10. The variations of impedance with annealing time for the four types of oils at frequency 1000 Hz and annealing temperatures: (a) 180˚C, (b) 160˚C, and (c) 140˚C, respectively.

Figure S11. The variations of acid value with annealing time for all oils annealed at temperatures：(a) 180˚C, (b) 160˚C, and (c) 140˚C, respectively.

* Corresponding author e-mail: sbLee@mx.nthu.edu.tw

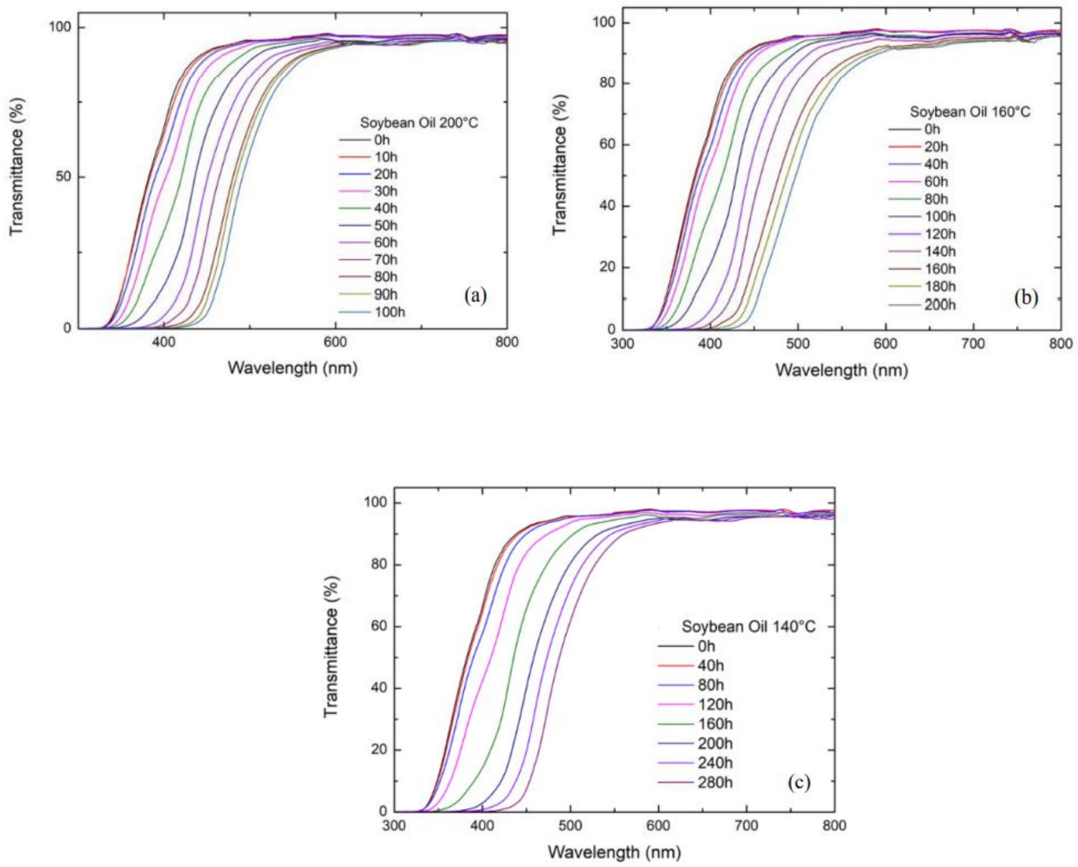

Figure S1. The variations of UV-Vis transmittance versus wavelength for soybean oils annealed for different times at temperatures: (**a**) 200°C, (**b**) 160°C, and (**c**) 140°C.

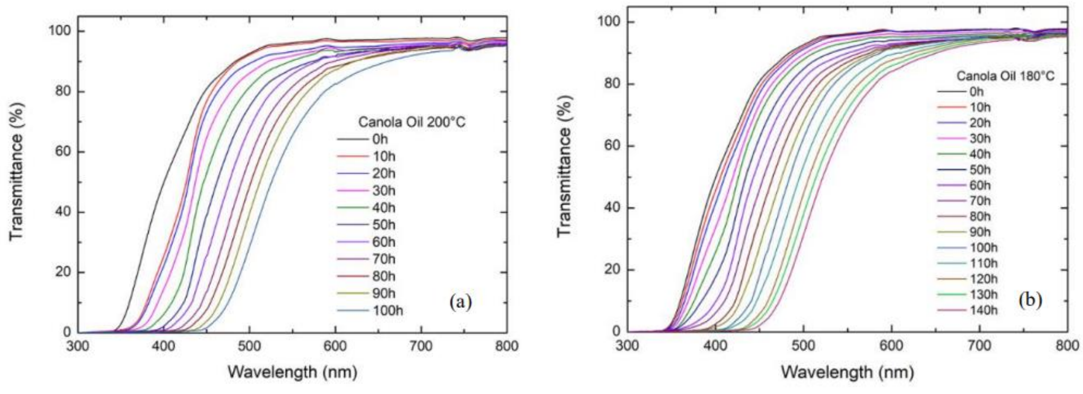

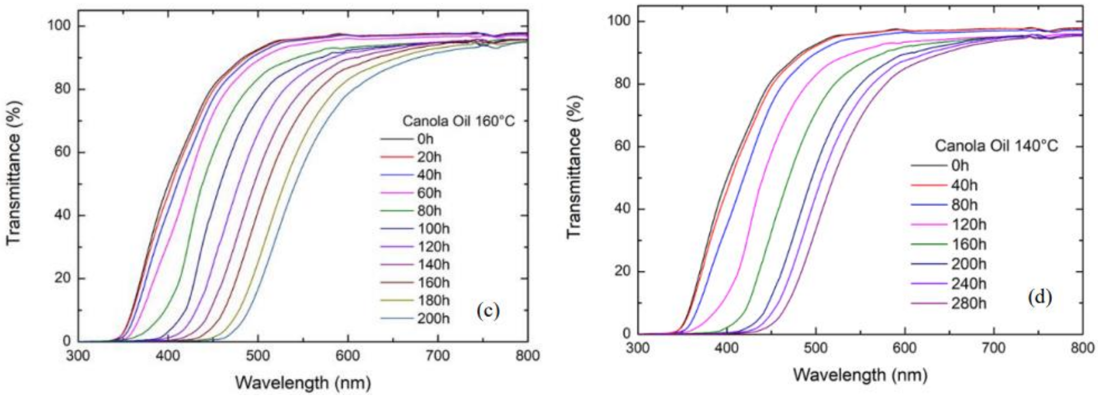

Figure S2. The variations of UV-Vis transmittance versus wavelength for canola oils annealed for different times at temperatures: (**a**) 200°C, (**b**) 180°C, (**c**) 160°C, and (**d**) 140°C

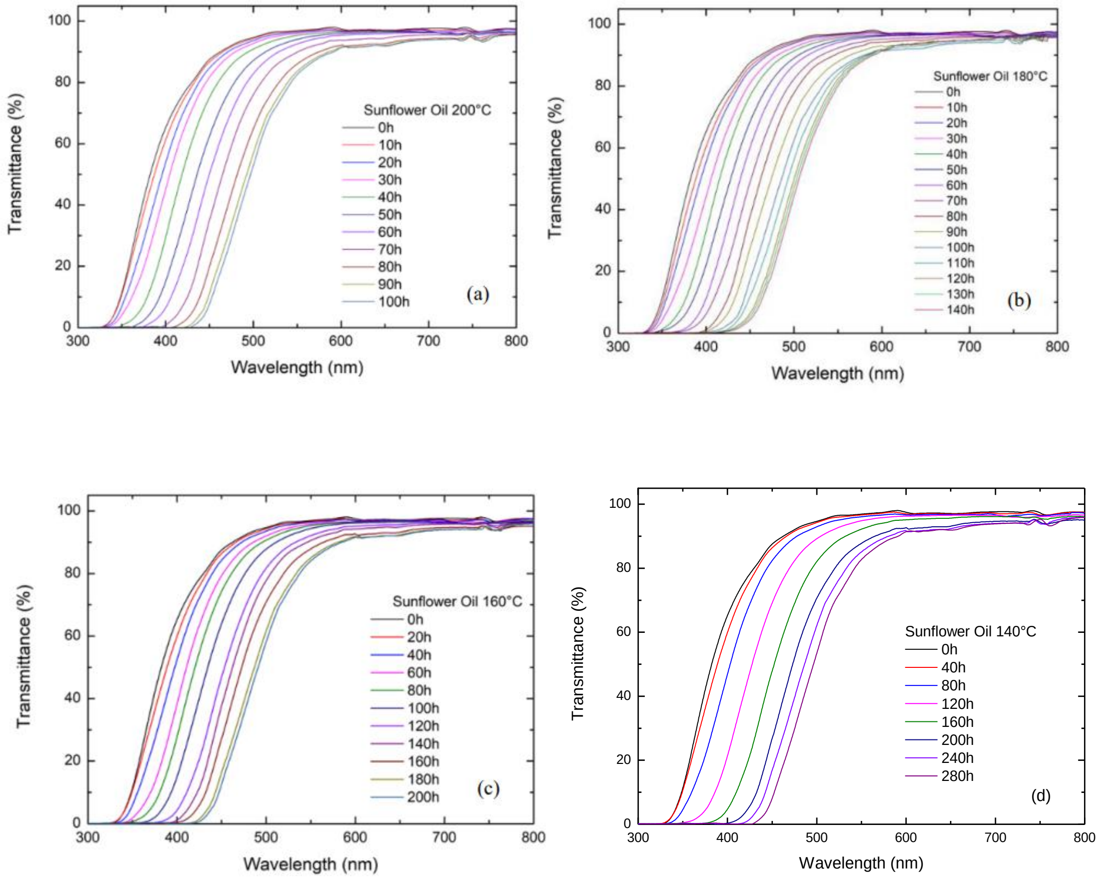

Figure S3. The variations of UV-Vis transmittance versus wavelength for sunflower oils annealed for different times at temperatures: (**a**) 200°C, (**b**) 180°C, (**c**) 160°C, and (**d**) 140°C.

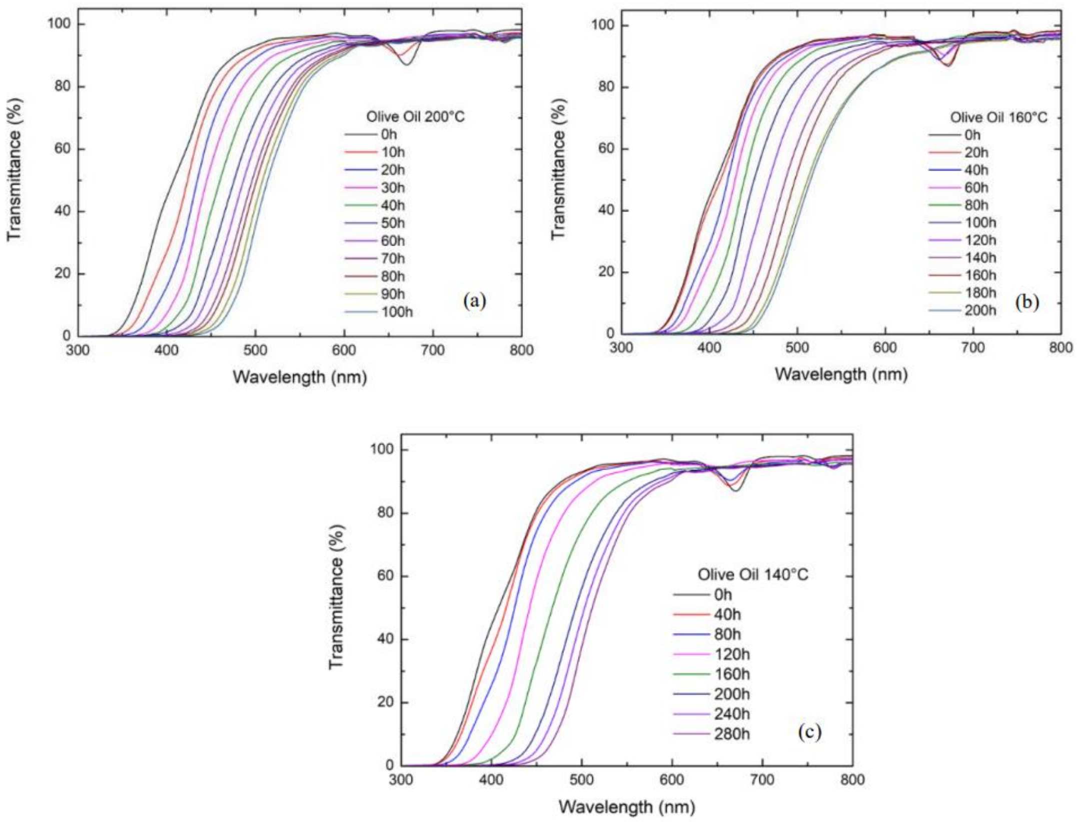

Figure S4. The variations of UV-Vis transmittance versus wavelength for olive oils annealed for different times at temperatures: (**a**) 200°C, (**b**) 160°C, and (**c**) 140°C

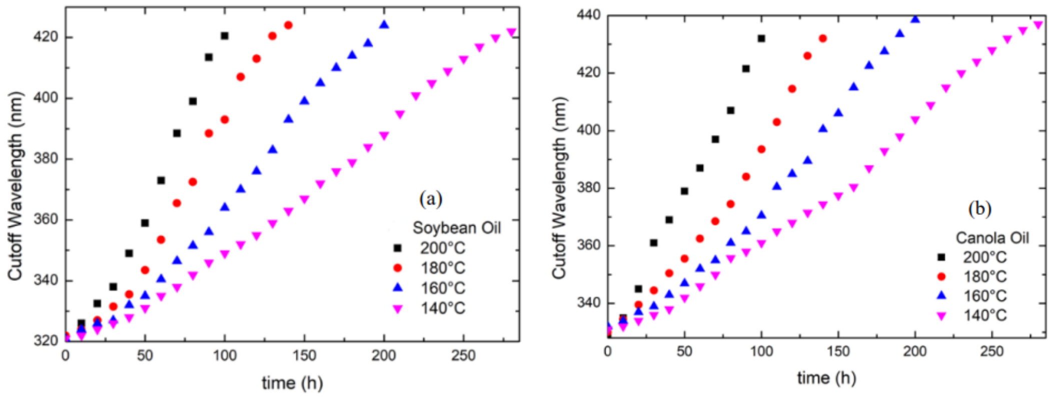

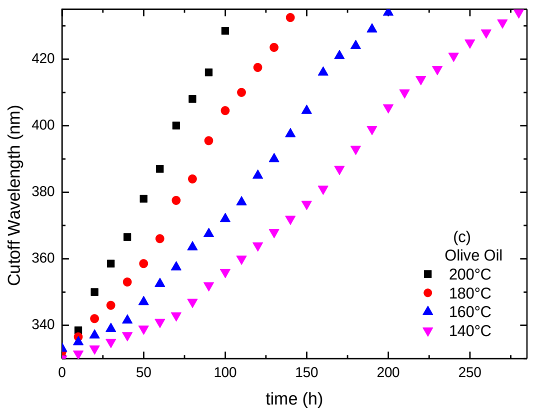

Figure S5. The variations of cutoff wavelength with annealing time at different annealing temperatures for (**a**) soybean oil, (**b**) canola oil, and (**c**) olive oil.

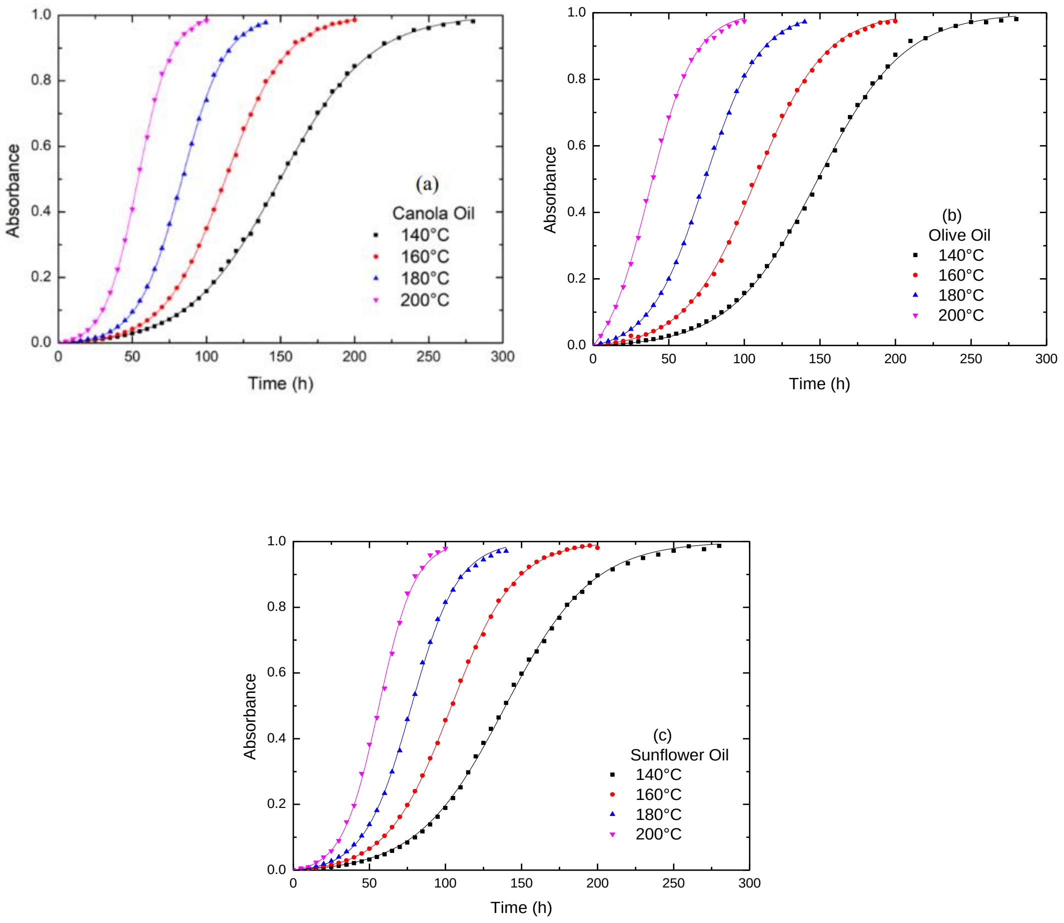

Figure S6. The variations of absorbance with time at different annealing temperatures: (**a**) soybean oil, (**b**) olive oil, and (**c**) sunflower oil.

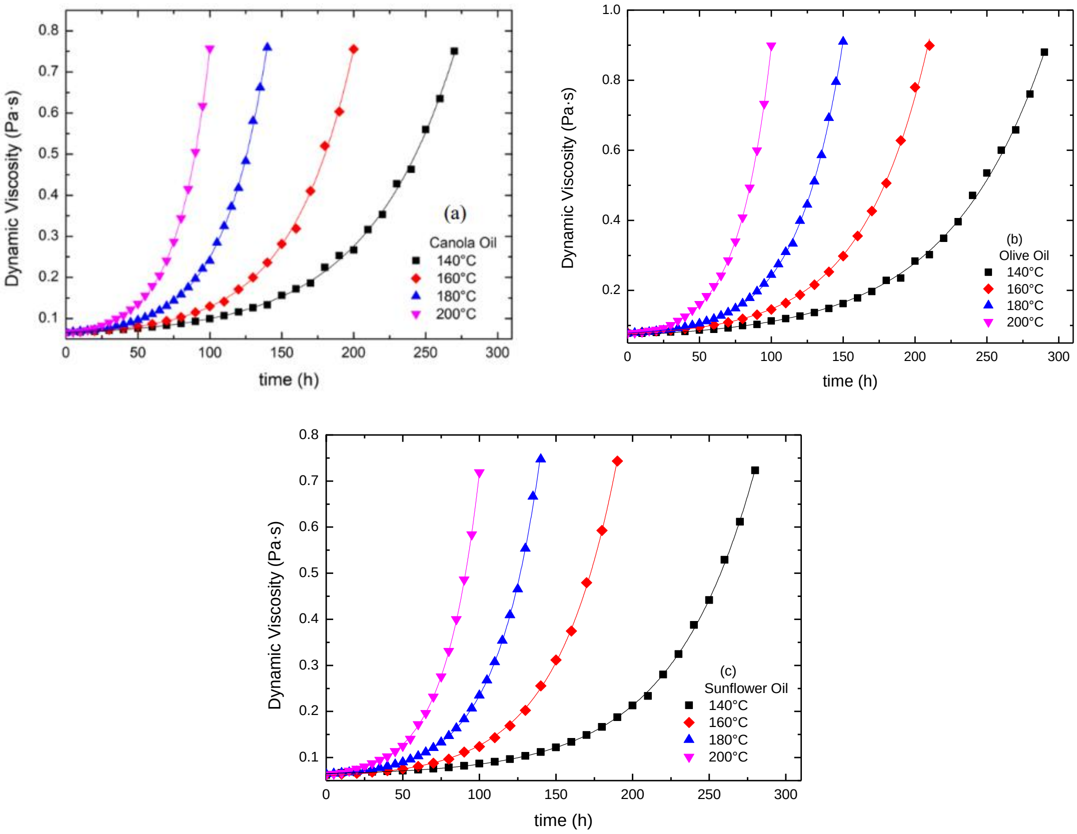

Figure S7. The variations of dynamic viscosity with annealing time at different annealing temperatures: (**a**) canola oil, (**b**) olive oil, and (**c**) sunflower oil.

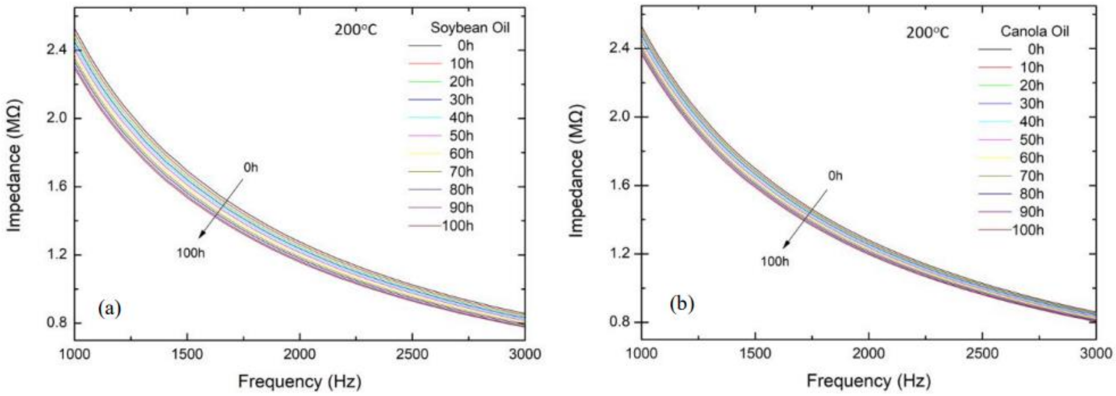

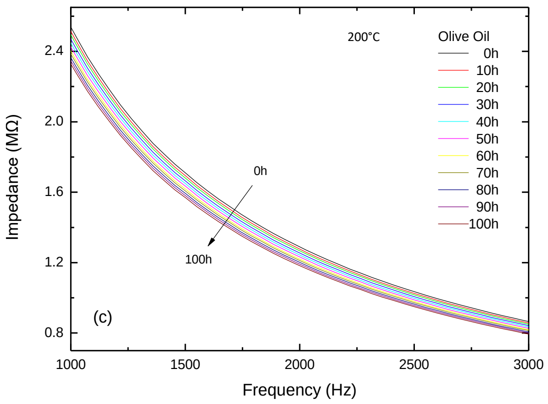

Figure S8. The variations of electrical impedance with the frequency for (**a**) soybean oil, (**b**) canola oil, and (**c**) olive oil annealed at 200°C for different annealing times.

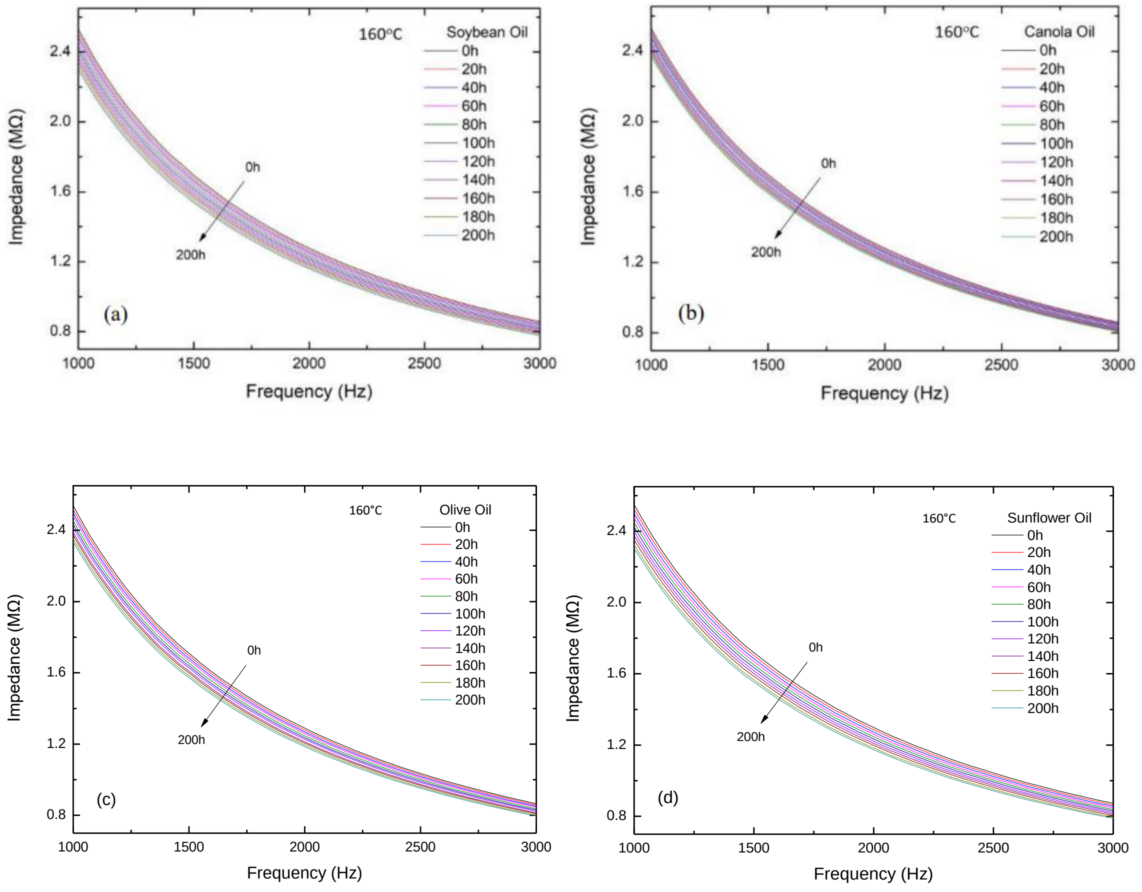

Figure S9. The variations of electrical impedance with the frequency for (**a**) soybean oil, (**b**) canola oil, (**c**) olive oil, and (**d**) sunflower oil annealed at 160°C for different annealing times.

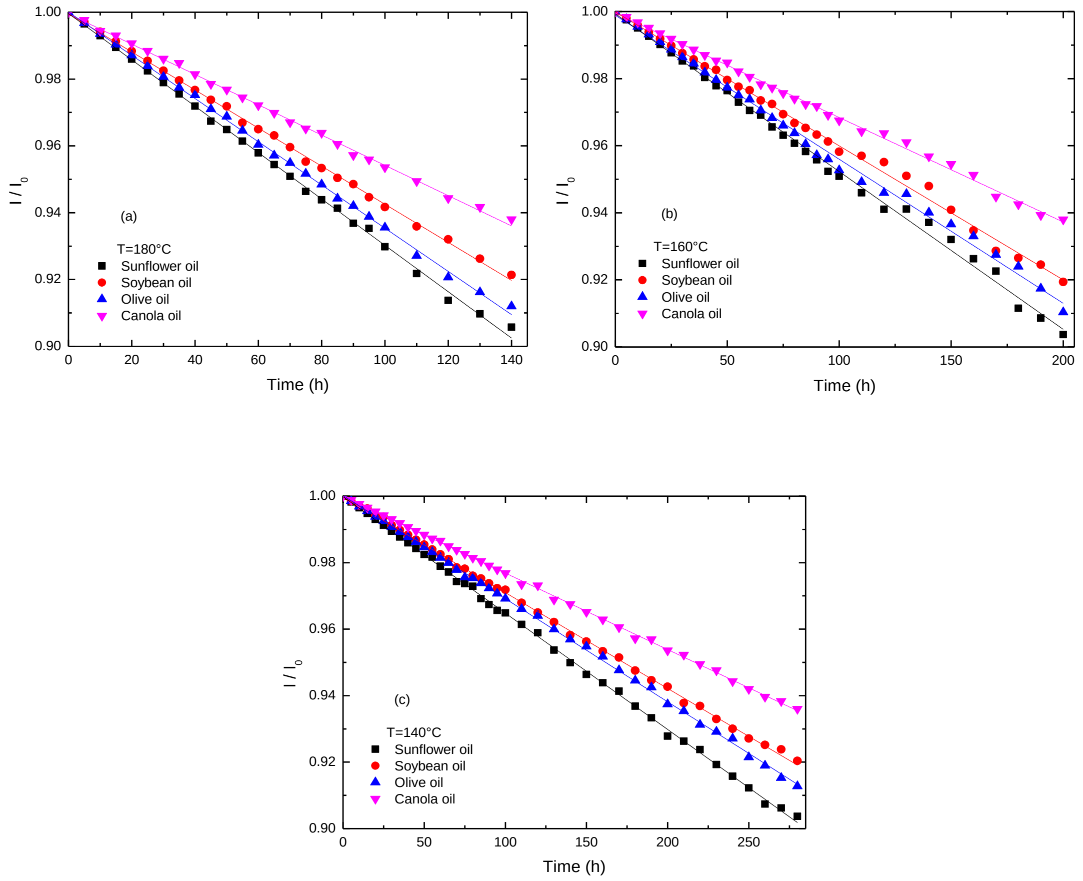

Figure S10. The variations of impedance with annealing time for the four types of oils at frequency 1000 Hz and annealing temperatures: (**a**) 180 °C, (**b**) 160 °C, and (**c**) 140 °C, respectively.

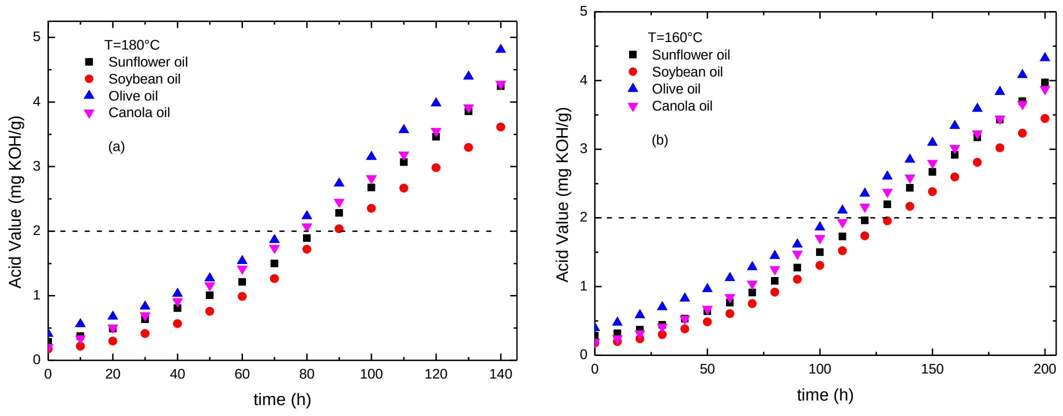

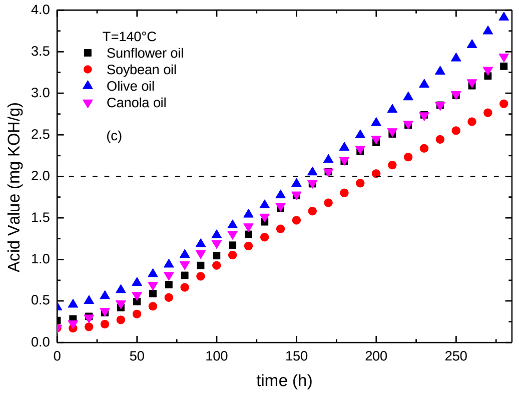

Figure S11. The variations of acid value with annealing time for all oils annealed at temperatures：(**a**) 180˚C, (**b**) 160˚C, and (**c**) 140˚C, respectively.

Table Captions

Table S1. Parameters α, A_0, and A_∞ to fit the absorbance data for canola oil at four annealing temperatures where R^2 is the confidence interval.

Table S2. Parameters α, A_0, and A_∞ to fit the absorbance data for olive oil at four annealing temperatures where R^2 is the confidence interval.

Table S3. Parameters α, A_0, and A_∞ to fit the absorbance data for sunflower oil at four annealing temperatures where R^2 is the confidence interval.

Table S4. The viscosity parameters φ_0, φ_R, and α_v to plot the solid lines in Figure S7(a) in Supplementary Material for the canola oil.

Table S5. The parameters φ_0, φ_R, and α_v to plot the solid lines in Figure S7(b) in Supplementary Material for the olive oil.

Table S6. The parameters φ_0, φ_R, and α_v to plot the solid lines in Figure S7(c) in Supplementary Material for the sunflower oil.

Table S7. The initial impedances of four oils at a frequency of 1000 Hz annealed at different temperatures.

Table S1. Parameters α, A_0, and A_∞ to fit the absorbance data for canola oil at four annealing temperatures where R^2 is the confidence interval.

temperature (°C)	α (h^{-1})	A_0	A_∞	R^2
200	0.089	0.0172	0.9826	0.996
180	0.067	0.0177	0.9823	0.998
160	0.049	0.0224	0.9776	0.997
140	0.033	0.0195	0.9802	0.998

Table S2. Parameters α, A_0, and A_∞ to fit the absorbance data for olive oil at four annealing temperatures where R^2 is the confidence interval.

Temperature (°C)	α (h^{-1})	A_0	A_∞	R^2
200	0.070	0.0115	0.9882	0.994
180	0.056	0.0201	0.9797	0.995
160	0.043	0.0214	0.9782	0.996
140	0.032	0.0294	0.9704	0.997

Table S3. Parameters α, A_0, and A_∞ to fit the absorbance data for sunflower oil at four annealing temperatures where R^2 is the confidence interval.

Temperature (°C)	α (h^{-1})	A_0	A_∞	R^2
200	0.084	0.0191	0.9808	0.997
180	0.065	0.0245	0.9700	0.996
160	0.048	0.0201	0.9797	0.997
140	0.035	0.0281	0.9715	0.996

Table S4. The viscosity parameters φ_0, φ_R, and α_v to plot the solid lines in Figure S7(a) in Supplementary Material for the canola oil.

Temperature (°C)	φ_0 (10^{-2} Pa.s)	φ_R (10^{-2} Pa.s)	α_v (10^{-2} h^{-1})	R^2
200	6.605	5.777	4.435	0.995
180	6.621	5.912	3.286	0.995
160	6.544	5.842	2.299	0.996
140	6.529	5.706	1.640	0.998

Table S5. The parameters φ_0, φ_R, and α_v to plot the solid lines in Figure S7(b) in Supplementary Material for the olive oil.

Temperature (°C)	φ_0 (10^{-2} Pa.s)	φ_R (10^{-2} Pa.s)	α_v (10^{-2} h^{-1})	R^2
200	7.638	6.636	4.417	0.998
180	7.840	7.144	3.197	0.996
160	7.703	6.789	2.159	0.998
140	7.309	6.174	1.476	0.998

Table S6. The parameters φ_0, φ_R, and α_v to plot the solid lines in Figure S7(c) in Supplementary Material for the sunflower oil.

Temperature (°C)	φ_0 (10^{-2} Pa.s)	φ_R (10^{-2} Pa.s)	α_v (10^{-2} h^{-1})	R^2
200	6.312	5.485	4.399	0.995
180	6.593	5.489	3.446	0.997
160	6.438	5.966	2.620	0.997
140	6.547	6.171	1.848	0.996

Table S7. The initial impedances of four oils at the frequency of 1000 Hz annealed at different temperatures.

Oil type / Temperature	Soybean	Canola	Olive	Sunflower
200 °C	2.464	2.466	2.472	2.480
180 °C	2.470	2.467	2.477	2.487
160 °C	2.469	2.470	2.478	2.490
140 °C	2.469	2.469	2.477	2.490
